# Chlorogenic Acid Inhibits BAFF Expression in Collagen-Induced Arthritis and Human Synoviocyte MH7A Cells by Modulating the Activation of the NF-*κ*B Signaling Pathway

**DOI:** 10.1155/2019/8042097

**Published:** 2019-05-22

**Authors:** Xiaohong Fu, Xilin Lyu, Han Liu, Dan Zhong, Zhizhen Xu, Fengtian He, Gang Huang

**Affiliations:** ^1^Department of Biochemistry and Molecular Biology, College of Basic Medical Sciences, Army Medical University (Third Military Medical University), Chongqing 400038, China; ^2^Department of Emergency, Southwest Hospital, Army Medical University, Chongqing 400038, China

## Abstract

B cell activating factor (BAFF), a member of the tumor necrosis factor (TNF) family, plays a critical role in the pathogenesis and progression of rheumatoid arthritis (RA). Chlorogenic acid (CGA) is a phenolic compound and exerts antiarthritic activities in arthritis. However, it is not clear whether the anti-inflammatory property of CGA is associated with the regulation of BAFF expression. In this study, we found that treatment of the collagen-induced arthritis (CIA) mice with CGA significantly attenuated arthritis progression and markedly inhibited BAFF production in serum as well as the production of serum TNF-*α*. Furthermore, CGA inhibits TNF-*α*-induced BAFF expression in a dose-dependent manner and apoptosis in MH7A cells. Mechanistically, we found the DNA-binding site for the transcription factor NF-*κ*B in the BAFF promoter region is required for this regulation. Moreover, CGA reduces the DNA-binding activity of NF-*κ*B to the BAFF promoter region and suppresses BAFF expression through the NF-*κ*B pathway in TNF-*α*-stimulated MH7A cells. These results suggest that CGA may serve as a novel therapeutic agent for the treatment of RA by targeting BAFF.

## 1. Introduction

Rheumatoid arthritis (RA) is a chronic inflammatory autoimmune disease and is characterized by hyperplasia of synovial lining cells and destruction of cartilage [1, 2]. Although the exact cause is not fully understood, it has been demonstrated that impaired apoptosis of fibroblast-like synoviocytes (FLSs) causes synovial hyperplasia, facilitating the destruction of cartilage in RA [3–5]. Moreover, RA-FLSs can produce abundant proinflammatory cytokines, such as B cell activating factor (BAFF), TNF-*α*, IL-6, and IL-1, which drive inflammation and induce cartilage destruction [6]. In particular, the expression of BAFF is induced by proinflammatory cytokines such as TNF-*α* and IL-6 [7, 8], and TNF-*α*-induced BAFF expression controls the survival of FLSs [9]. Therefore, the expression of BAFF plays a critical role in FLSs, which was associated with RA processes.

BAFF is originally known as a cytokine in B cell development, survival, and proliferation. The previous studies confirmed that it is produced not only by immune cells (B lymphocytes, monocytes, and macrophages) but also by nonimmune cells (prostate epithelium and FLS) [10–12]. Elevated levels of BAFF have been detected in serum and synovial tissue from patients with RA, and the levels of BAFF were positively correlated with the intensity of the local inflammatory response in RA patients [6, 13]. Furthermore, TNF-*α*-induced BAFF controls RA angiogenesis by regulating VEGF expression in synoviocytes [14, 15], and the mechanism by which FLSs induce class switch recombination (CSR) was BAFF-dependent [16, 17]. Consequently, BAFF may serve as a novel molecular target for treatment of RA [13, 18]. However, little has been known about the regulation of BAFF expression in RA-FLS. Recently, the phenolic compound chlorogenic acid (CGA) exerts anti-inflammatory activities in arthritis, and it has a regulatory role in gene expression [19–21].

The previous study demonstrated that CGA effectively controlled the total (CD3) and differentiated (CD4 and CD8) T cell count in adjuvant-induced arthritis rats [22]. CGA inhibited the expression of MMP-1, MMP-3, and MMP-13 while increasing TIMP-1 expression, at both the mRNA and protein levels in the osteoarthritis (OA) animal model [23, 24]. Moreover, CGA inhibited the proliferation of FLSs stimulated by IL-6 significantly, induced cell apoptosis notably, and suppressed the expression of key molecules in the JAK/STAT and NF-*κ*B signaling pathways [25, 26]. These studies indicate that CGA may be considered as a possible candidate agent in the treatment of arthritis. However, it is not clear whether the anti-RA effect of CGA is associated with the regulation of BAFF expression in collagen-induced arthritis (CIA) mice and in MH7A synovial cells. In the present study, we found that CGA markedly ameliorated arthritis progression in CIA mice in a dose-dependent manner, which was accompanied with the inhibition of BAFF production as well as the decrease of TNF-*α*. We also found that CGA significantly inhibited TNF-*α*-induced BAFF expression possibly through the suppression of NF-*κ*B signaling pathways *in vitro*. These studies indicate that the repression of BAFF production may be a novel mechanism by which CGA improves RA.

## 2. Materials and Methods

### 2.1. CIA Induction and Treatment

Male DBA/1J mice were purchased from the Laboratory Animal Center of China. All mice were used at 7–9-week old and maintained under specific pathogen-free (SPF) conditions. Animal studies complied with the World Medical Association Declaration of Helsinki and were approved by the Animal Care Committee of the Third Military Medical University. DBA/1J mice were immunized with chicken type II collagen (CII) (Chondrex, Redmond, USA) as described previously [27]. To examine the antiarthritic effect of the CGA (Sigma, MO, USA), mice with CIA were injected intraperitoneally with CGA (30 mg/kg or 60 mg/kg) daily from day 28 after the first immunization. CIA control mice received vehicle alone. The severity of arthritis in all four paws of mice was evaluated using a five-degree score (as presented by Rosloniec et al.) to consider the different combinations of inflamed groups of joints and to reduce the influence of the subjectivity on the investigator's evaluation [28].

### 2.2. Measurement of Proinflammatory Cytokine Levels

For the analysis of serum cytokine levels, BAFF was quantified using the mouse BAFF Quantikine ELISA kit (R&D Systems, MN, USA) according to the manufacturer's protocol. The Mouse Cytokine/Chemokine Magnetic Bead Panel (Cat. # MCYTOMAG-70K, Millipore, MA, USA) was used for the TNF-*α* assay according to the manufacturer's instructions using a Luminex platform.

### 2.3. Histological Analysis

The hind paws were fixed in 10% neutral-buffered formalin, then decalcified in 15% EDTA, and embedded in paraffin for histopathological analysis. The sections (5 *μ*m) were stained with hematoxylin and eosin (HE) according to standard methods. The joint pathology was examined and scored as previously described [29].

### 2.4. Cell Viability Assay

The cell viability was assayed using a CCK-8 kit (Dojindo, Shanghai, China). MH7A cell was cultured in DMEM supplemented with 15% (*v*/*v*) fetal bovine serum (FBS) at 37°C in 5% CO_2_. Briefly, MH7A cells (1 × 10^4^ cells/ml, 200 *μ*l per well) were seeded in triplicate in 48-well flat-bottom plates for 12 h, followed by treatment with various concentrations of CGA (20, 50, 80, and 100 *μ*M) for 24 h, taking 0.1% DMSO as the vehicle control. Then, 20 *μ*l of CCK-8 solution was added to each well, and the cells were incubated at 37°C for 1.5 h. Subsequently, the OD value at 450 nm was measured with a microplate reader (Molecular Devices, Sunnyvale, CA, USA), and cell viability (%) was calculated compared with the control group. The experiment was repeated three times in triplicate.

### 2.5. Flow Cytometry

MH7A cells were stimulated with 20 ng/ml TNF-*α* (PeproTech, Princeton, USA) for 4 h and then treated with different doses of CGA for 24 h. Subsequently, cells were trypsinized and collected for the detection of apoptotic cells using an Annexin V-FITC Apoptosis Detection kit (Cat# 556547, BD Biosciences, USA). Briefly, MH7A cells were washed twice with cold PBS at 4°C and resuspended in 100 *μ*l binding buffer. After stained with FITC-conjugated Annexin V and propidium iodide (PI), cells were incubated for 15 min at room temperature and then analyzed by flow cytometry MultiCycle AV Phoenix Flow Systems (San Diego, CA, USA). All experiments were performed three times.

### 2.6. RNA Purification and Analysis

Total RNAs were extracted with the TRIzol reagent (Invitrogen, Carlsbad, CA, USA), and cDNA was synthesized from total RNA using MMLV reverse transcriptase (Invitrogen, Carlsbad, CA, USA) and oligo (dT). Real-time quantitative PCR (qPCR) was performed using the SYBR Green Mix on iQ5 system (Bio-Rad, Hercules, CA, USA) according to the manufacturer's protocol. The following primers were used for human BAFF, 5′-TTCCATGGCTTCTCAGCTTT-3′ (forward primer) and 5′-GTCCCATGGCGTAGGTCTTA-3′ (reverse primer), and for human *β*-actin, 5′-GTGAAGGTGACAGCAGTCGGTT-3′ (forward primer) and 5′-GAAGTGGGGTGGCTTTTAGGA-3′ (reverse primer). Relative expression was calculated with normalization to *β*-actin values by using the 2^−∆∆Ct^ method. Reactions were repeated minimum of three times in triplicate.

### 2.7. Western Blot Analysis

MH7A cells were stimulated with 20 ng/ml TNF-*α* for 4 h and then treated with different doses of CGA for 24 h. Dexamethasone (DEX) sodium phosphate (MedChemExpress, USA) treats the cells for 24 h as a positive control. Subsequently, proteins were extracted, and concentrations were determined using the BCA kit (Beyotime, China). Subsequently, protein from each sample was separated by 12% SDS-PAGE and transferred to a polyvinylidene fluoride membrane (Millipore Co., Bedford, MA). After blocking with 5% fat-free dry milk in Tris-buffered saline with Tween-20 (TBST) (10 mM Tris-HCl, pH 7.6; 150 mM NaCl; and 0.5% Tween 20) for 1 h, the membranes were separately incubated with the primary antibodies against p65 (1 : 500), BAFF (1 : 500), I*κ*B-*α* (1 : 200), pI*κ*B-*α* (1 : 200), and *α*-tubulin (1 : 500) overnight at 4°C. The mouse anti-BAFF (sc-80337), mouse anti-I*κ*B-*α* (sc-1643), mouse anti-p-I*κ*B-*α* (sc-8404), mouse anti-*α*-tubulin (sc-58667), and rabbit anti-NF-*κ*B p65 (sc-109) antibodies were purchased from Santa Cruz Biotechnology (Santa Cruz, CA, USA). After washing, the membranes were separately incubated with the corresponding horseradish peroxidase-conjugated secondary antibodies (1 : 5000; Zhongshan Biotechnology, Beijing, China) for 1 h at room temperature. Subsequently, the membranes were washed, and the signals were visualized with SuperSignal West Dura Extended Duration Substrate (Pierce, Rockford, IL, USA). Each experiment was performed three times.

### 2.8. Construction of Reporter Plasmids and Luciferase Assays

Different lengths of the human *BAFF* promoter region were amplified by PCR using MH7A cell genomic DNA as a template; then, the fragments including (-929 to +50) and (-750 to +50) were separately cloned into the pGL3-basic vector (Promega) using T4 DNA ligase (Takara) after digestion with *KpnI/BgIII* (Takara), and the resulting reporter plasmids were named, respectively, as pBAFF/930 and pBAFF/750. The plasmid containing a mutation at the NF-*κ*B-binding site (located in the BAFF promoter from −874 to −858) was constructed and verified as described [30].

MH7A cells were seeded in 48-well plates (1 × 10^4^ cells/well), grown to 70%-80% confluence; then, the cells were transiently transfected with the above luciferase reporter expression vectors, respectively, using Lipofectamine 3000 (Invitrogen, Carlsbad, CA, USA). Six hours later, the cells were stimulated with 20 ng/ml TNF-*α* for 4 h and then treated with CGA (50 and 100 *μ*M) for 20 h. Subsequently, the cell extracts were prepared, and the luciferase activity was determined using the dual-luciferase reporter system (Promega, Madison, Wisconsin, USA) according to the manufacturer's instruction. Transfection experiments were carried out three times in triplicate.

### 2.9. Electrophoretic Mobility Shift Assay (EMSA)

EMSA was performed using the LightShift Chemiluminescent EMSA kit (Pierce, Rockford, IL, USA), according to the manufacturer's instructions. Briefly, the biotin-labeled probes of the NF-*κ*B response element (−874 to −858, 5′-AACTGGGGAATGTCCAG-3′) were incubated with nuclear extracts for 20 min at room temperature. The reaction samples were electrophoresed in a 6% nondenaturing polyacrylamide gel in a 0.5x Tris-borate-EDTA buffer. After being transferred to the nylon membrane, the biotin-labeled probes were detected by chemiluminescence.

### 2.10. Statistical Analysis

All data were expressed as means ± standard deviation (SD). Comparisons between two groups were made with unpaired Student's *t*-test. Comparisons among 3 or more groups were analyzed by one-way analysis of variance (ANOVA) followed by Tukey-Kramer post hoc analysis. In all cases, *P* < 0.05 was regarded as statistically significant.

## 3. Results

### 3.1. Effect of CGA Treatment on CIA Evolution

**Figure 1:**
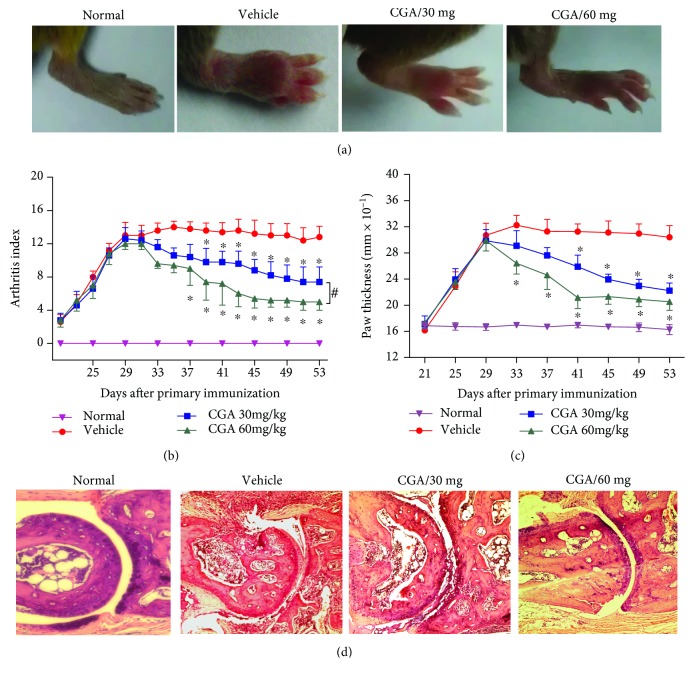
CGA attenuates the severity of CIA in mice. DBA/1J mice were immunized with 100 *μ*g of type II collagen on day 1. CGA (30 mg/kg and 60 mg/kg) and control vehicles (DMSO) were intraperitoneally injected daily from day 28 after the first immunization. Data are expressed as means ± SD for each group. ^∗^*P* < 0.05 vs. the vehicle-treated group, ^#^*P* < 0.05 vs. the CGA (30 mg/kg)-treated group. (a) Macroscopic observation of the hind feet of normal, vehicle-treated, and CGA-treated mice. (b, c) Intraperitoneal injection with CGA or vehicle from day 28 to day 53; the mean arthritis index (b) and paw thickness (c) were calculated at the indicated time points. (d) Hematoxylin and eosin staining (original magnification was ×10) of paw sections from each group of mice.

Firstly, the CIA mouse model was used to evaluate the anti-inflammatory activities of CGA. As shown in (Figure 1(a)), treatment the CIA mice with CGA (30 mg/kg and 60 mg/kg) or vehicle control from day 28 to day 53, the macroscopic observation of joint swelling was significantly higher in CIA control mice than in CGA-treated mice. Furthermore, the arthritic index was markedly suppressed in CGA-treated mice in a dose-dependent manner (Figure 1(b)). Consistent with this result, the footpad thickness in CIA mice treated with CGA obviously less than that in CIA control mice (Figure 1(c)). Histological evaluation of joint sections at day 53 revealed signs of severe arthritis and bone erosion in CIA control mice. However, CGA treatment remarkably attenuated the histological damage in the joint sections (Figure 1(d)). These data showed that the administration of CGA could suppress the progression of CIA in mice.

### 3.2. CGA Inhibits Proinflammatory Cytokine Production in CIA Mice

**Figure 2:**
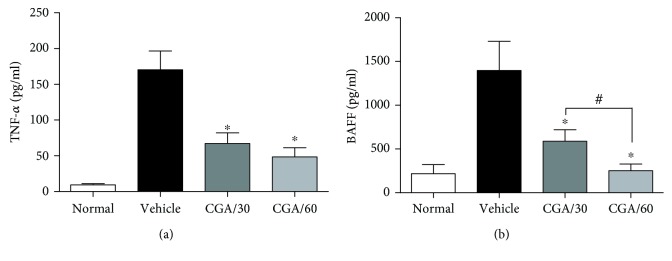
CGA inhibits proinflammatory cytokine production in CIA mice. (a) Serum concentrations of TNF-*α* from each group of mice were measured by Luminex on day 53 after the first immunization. (b) Serum from normal, vehicle-treated, and CGA-treated CIA mice was harvested, and the BAFF production was analyzed by ELISA. ^∗^*P* < 0.05 vs. the vehicle-treated group and ^#^*P* < 0.05 vs. the CGA (30 mg/kg)-treated group.

To determine whether CGA suppressed the progression of arthritis was involved in regulation of proinflammatory cytokines expression in CIA mice, the serum was collected and the level of TNF-*α* and BAFF were analyzed. Consistent with the observation of ameliorated clinical parameters (Figure 1), the serum level of TNF-*α* and BAFF was elevated and CGA treatment significantly reduced the serum TNF-*α* and BAFF level (Figures 2(a) and 2(b)) in CIA mice. These results indicated that the inhibition of the TNF-*α* and BAFF level was associated with CGA antiarthritis in CIA mice. It also suggested that CGA attenuates the severity of arthritis and suppresses the BAFF expression which may be associated with the repression of TNF-*α* production.

### 3.3. Effects of CGA on MH7A Cell Viability and Apoptosis

**Figure 3:**
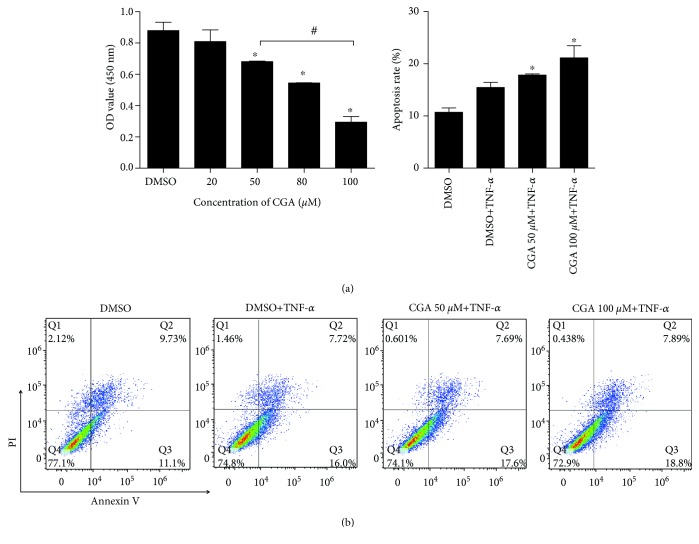
Effects of CGA on MH7A cell viability and apoptosis. (a) MH7A cells were treated with different concentrations of CGA (0, 20, 50, 80, and 100 *μ*M) for 24 h; the viability was determined by CCK-8 assay. (b) CGA-induced apoptosis of MH7A cells stimulated with TNF-*α*. Annexin V-PI-stained cells were observed using a flow cytometer after 24 h incubation. Histograms showed the statistical analysis for the ratio of apoptotic cells. Values are presented as the means ± SD (*n* = 3). ^∗^*P* < 0.05 vs. DMSO and ^#^*P* < 0.05 vs. CGA 50 *μ*M+TNF-*α* treated.

To assess whether CGA could inhibit human rheumatoid FLS proliferation, the effect of CGA on MH7A cell viability was determined using CCK-8. As shown in Figure 3(a), CGA inhibited the proliferation of MH7A cells in a dose-dependent manner compared with the corresponding controls (*P* < 0.05) and CGA with the dose of 50 *μ*M and 100 *μ*M was utilized in the following experiments. To further evaluate the pathological significance of CGA during RA progression, we treated MH7A cells with TNF-*α* and CGA and then detected the ratios of apoptosis of MH7A cells by Annexin V-PI staining using a flow cytometer. As shown in Figure 3(b), CGA had a significant effect on the apoptosis of MH7A cells induced by TNF-*α*. Thus, these results indicated that CGA might precipitate the apoptosis of FLSs induced by inflammatory cytokines for inhibiting inflammatory proliferation of FLS.

### 3.4. CGA Suppresses TNF-*α*-Induced BAFF Expression in MH7A Cells

**Figure 4:**
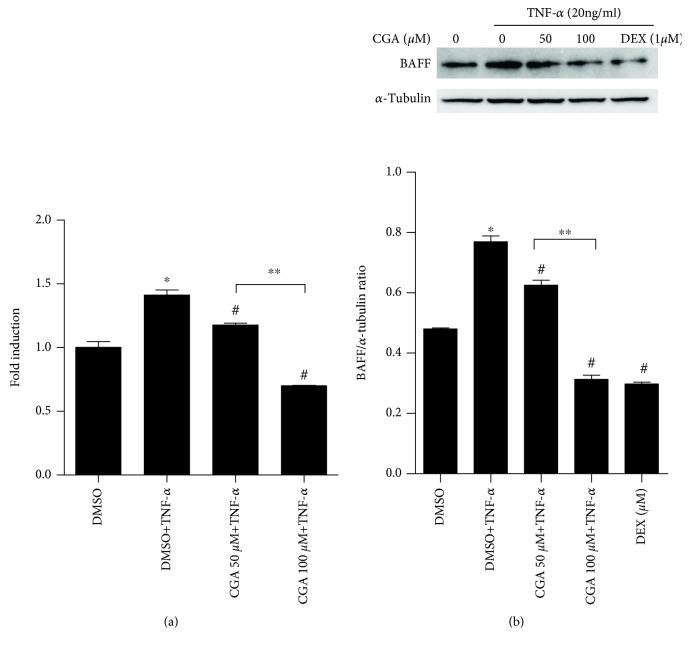
CGA suppresses TNF-*α*-induced BAFF expression in MH7A cells. Cells were stimulated with 20 ng/ml TNF-*α* for 4 h and then treated with CGA (50 and 100 *μ*M) for 20 h; the BAFF expression was analyzed by real-time quantitative PCR (a) and Western blot (b). DEX was used as a positive control, and *α*-tubulin was used as a loading control. The densitometry analysis for Western blot was provided. ^∗^*P* < 0.05 vs. DMSO, ^#^*P* < 0.05 vs. TNF-*α* treated, and ^∗∗^*P* < 0.05 vs. CGA 50 *μ*M+TNF-*α* treated.

As shown in Figure 2, the changes of TNF-*α* were paralleled with BAFF, which can induce BAFF expression *in vivo*. To elucidate whether CGA suppresses TNF-*α*-induced BAFF expression *in vitro*, MH7A cells were pretreated with TNF-*α* before exposing to CGA, DEX, or vehicle DMSO for 24 h. As shown in Figures 4(a) and 4(b), TNF-*α* (20 ng/ml) dramatically induced BAFF mRNA (Figure 4(a)) and protein (Figure 4(b)) expression in MH7A cells, which was markedly reduced by CGA treatment in a dose-dependent manner. In addition, the expression of BAFF was markedly reduced in the positive control of DEX (Figure 4(b)). These findings indicated that CGA repressed TNF-*α*-induced BAFF expression at both mRNA and protein levels.

### 3.5. CGA Inhibits the Transcriptional Activity of the BAFF Promoter

**Figure 5:**
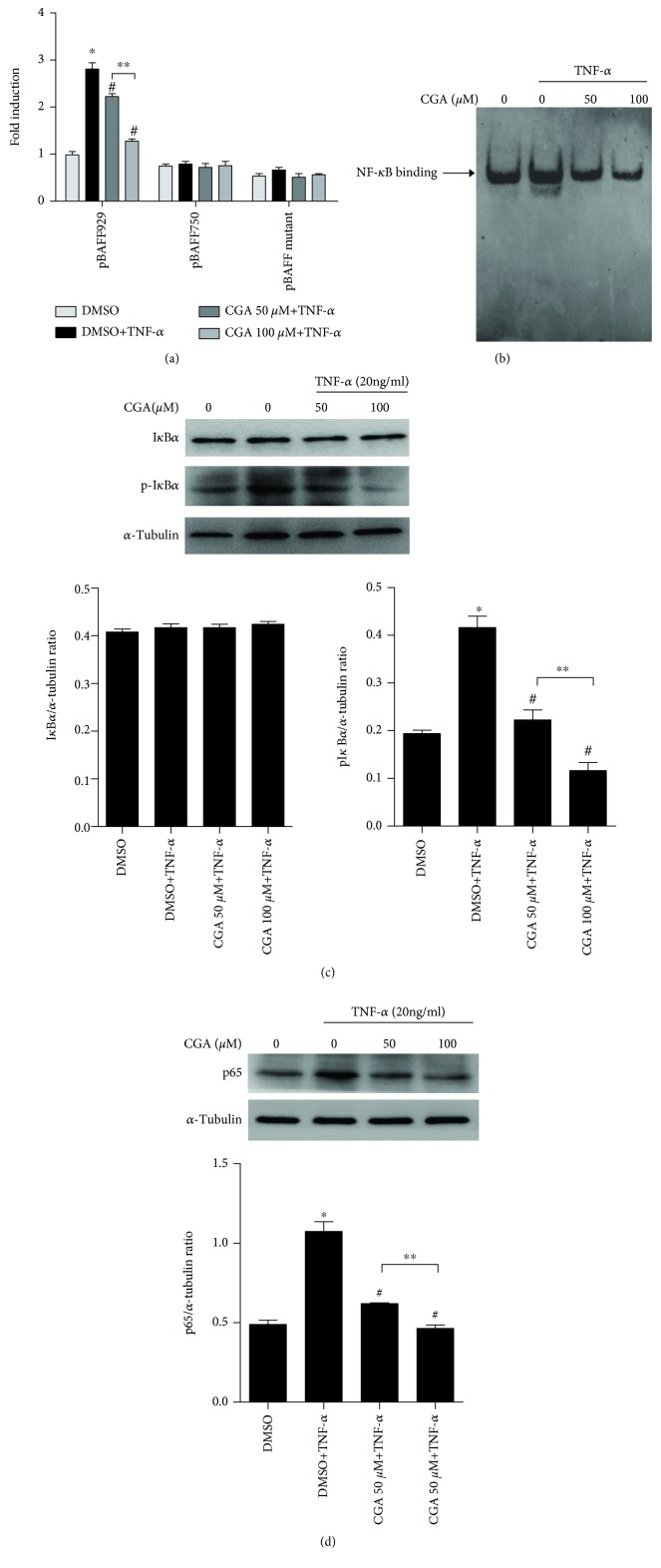
CGA alleviates the binding activity of NF-*κ*B to the BAFF promoter by suppressing the NF-*κ*B pathway. (a) MH7A cells were transiently transfected with the luciferase reporter plasmid pBAFF/929, pBAFF/750, or pBAFF/mutant, followed by stimulation with 20 ng/ml TNF-*α* for 4 h, and then treatment with CGA (50 and 100 *μ*M) for 20 h. The luciferase assay was performed. Data were means ± SD from three experiments in triplicate. (b) Cells were treated as above; the nuclear extracts were prepared, and EMSA was performed. (c, d) MH7A cells were treated as above, and then, I*κ*B*α*, phospho-I*κ*B*α* (p-I*κ*B*α*), and p65 were examined by Western blot. *α*-Tubulin was used as a loading control. The densitometry analysis for Western blot was provided. ^∗^*P* < 0.05 vs. DMSO, ^#^*P* < 0.05 vs. TNF-*α* treated, and ^∗∗^*P* < 0.05 vs. CGA 50 *μ*M+TNF-*α* treated.

Suppression of BAFF mRNA expression by CGA (Figure 4) suggested that CGA modulated BAFF expression at the transcriptional level and exerted its inhibitory activity on the BAFF gene promoter. Our previous study has shown that there was a NF-*κ*B-binding site located in the BAFF promoter region (−874 to −858) and thus regulated BAFF expression [31]. To further verify whether CGA-mediated suppression of BAFF expression was via interfering with NF-*κ*B signaling, report assay was performed in MH7A cells after treatment as above. As shown in Figure 5(a), treatment with CGA dramatically reduces TNF-*α*-induced pBAFF/929 (BAFF promoter region −929 to +50) activation, but TNF-*α* and CGA treatment had no influence on the luciferase reporter activity of pBAFF/750 (BAFF promoter region −750 to +50), indicating that the DNA fragment (−929 to −750) in the BAFF promoter region might contain the response element to TNF-*α* and CGA. Furthermore, the mutation of the NF-*κ*B-binding site (−874 to −858) in pBAFF/929 also significantly suppressed the activity of the BAFF promoter region and resulted in failure in response to TNF-*α* and CGA. These data suggested that CGA repressed the BAFF expression which may be associated with NF-*κ*B binding to the BAFF promoter.

### 3.6. CGA Alleviates the Binding of NF-*κ*B to the BAFF Promoter by Suppressing the NF-*κ*B Pathway

To investigate whether CGA inhibition of the TNF-*α*-induced BAFF expression was via interfering with the NF-*κ*B-binding site, EMSA was performed with the nuclear extracts derived from MH7A cells after treatment with CGA. As shown in Figure 5(b), TNF-*α* enhanced the binding of NF-*κ*B to the *BAFF* gene promoter and CGA reduced the binding activity. Subsequently, to investigate the mechanism by which CGA inhibited the TNF-*α*-induced BAFF expression in MH7A cells, we assessed I*κ*B*α*, phosphorylated I*κ*B*α*, and p65 to determine whether the NF-*κ*B signaling molecules control the BAFF expression. As shown in Figure 5(c), the p-I*κ*B*α* increased significantly in TNF-*α*-treated and CGA markedly inhibited TNF-*α*-induced phosphorylation of I*κ*B*α* in a dose-dependent manner. In addition, the protein level of p65 in MH7A was consistent with the p-I*κ*B*α* expression (Figure 5(d)). These results demonstrated that CGA may repress TNF-*α*-induced BAFF expression via negatively interfering with NF-*κ*B signaling.

## 4. Discussion

RA is characterized by synovial hyperplasia and destruction of cartilage and bone [31]. Synovial FLSs in patients with RA secrete abundant inflammatory cytokines and proteases which contribute to cartilage destruction [32]. The previous study confirmed that FLSs can invade the synovium through pores in the cortical bone in CIA mice [33]. Furthermore, when there is disrupted balance between cell proliferation, survival, and death, the FLSs in RA will be notably increased. The environment of the synovial in RA is beneficial to FLS survival because of the inhibition of apoptosis [34, 35]. Therefore, these studies suggested that targeting FLSs should be an important component of RA treatment. In this study, we found that CGA could suppress the proliferation of FLS. We supposed that CGA suppressed the proliferation of FLSs in synovium by inducing FLS cell apoptosis. The results showed that the apoptotic cells obviously increased when cells treated with CGA.

As mentioned in Introduction, inflammatory cytokines play a critical role in the pathogenesis of RA. Among these cytokines, TNF-*α* is known to induce the generation of other inflammatory cytokines in RA, such as BAFF [6, 36]. In the present study, we found that CGA has a therapeutic effect on ongoing arthritis, which effectively ameliorate the severity of collagen-induced arthritis in DBA/1J mice. This suppressive effect was accompanied with decrease of TNF-*α* and BAFF production *in vivo*. Furthermore, we have further shown for the first time that CGA downregulation of TNF-*α* induced the BAFF expression in a dose-dependent manner *in vitro*. Therefore, these *in vivo* and *in vitro* results indicated that the anti-inflammatory property of CGA is associated with the inhibition of BAFF expression, which may be helpful in illustrating the detailed antiarthritis mechanisms of CGA and may be beneficial in developing a new strategy for the treatment of RA.

The previous studies demonstrated that CGA has biochemical functions including anti-inflammatory, antioxidative, and anticarcinogenic effects [25, 26]. In order to investigate the molecular mechanism of CGA inhibition of the TNF-*α*-induced BAFF expression in MH7A, we next explored the regulating effects of CGA on the activation of the NF-*κ*B signaling pathway. The previous studies confirmed that there was a NF-*κ*B-binding site located in the BAFF promoter region (−874 to −858), and the activation of the NF-*κ*B can regulate BAFF expression [27, 30]. NF-*κ*B is a transcription factor that is involved in cell survival, proliferation, and inflammation. Increased NF-*κ*B activity contributes to the chronic inflammation characteristic of RA. NF-*κ*B proteins commonly form heterodimers (p50 and p65) and normally combine with I*κ*B*α* to form a cytoplasmic complex, which inhibits its entry into the nucleus. Upon stimulation, I*κ*B*α* was phosphorylated and then the p65 subunit of NF-*κ*B translocated to the nucleus. The NF-*κ*B in the nucleus bind to the promoter regions and induce the target gene expression. In the present study, we demonstrated that CGA significantly inhibited TNF-*α*-induced phosphorylation of I*κ*B*α* and expression of NF-*κ*B p65, which result to the inhibition of the DNA-binding activity of NF-*κ*B to the BAFF promoter region. These results are consistent with the EMSA assay. The above data suggested the molecular mechanism for CGA antiarthritis at least in part through inhibiting the NF-*κ*B signaling pathway and consequently BAFF expression. However, the mechanism by which CGA inhibits the BAFF expression has not confirmed *in vivo*, and the detailed mechanism by which CGA suppresses the phosphorylation of I*κ*B*α* is still unknown.

In summary, although it has not been verified all possible target molecules for CGA anti-RA in FLSs, our finding suggested that BAFF could be inhibited by CGA through the NF-*κ*B signaling pathway. These results may provide a novel role for BAFF in synovial cells other than B lymphocytes. These data also suggested that CGA may serve as a novel therapeutic agent by targeting BAFF in the treatment of RA.

## Data Availability

The data used to support the findings of this study are included within the article.
